# Effects of quinoa on cardiovascular disease and diabetes: a review

**DOI:** 10.3389/fnut.2024.1470834

**Published:** 2024-10-04

**Authors:** He Zhang, Ruiqi Li

**Affiliations:** ^1^Xiyuan Hospital, China Academy of Chinese Medical Sciences, Beijing, China; ^2^National Clinical Research Center for Chinese Medicine Cardiology, Beijing, China; ^3^The Third Affiliated Hospital of Beijing University of Chinese Medicine, Beijing, China

**Keywords:** quinoa, cardiovascular disease, diabetes, efficacy, review

## Abstract

Quinoa is an annual dicotyledonous plant belonging to the genus *Chenopodiaceae*. As a functional healthy food with outstanding nutritional value, quinoa contains not only a balanced proportion of amino acids but also higher contents of protein, unsaturated fatty acids, vitamins, and minerals (K, P, Mg, Ca, Zn, and Fe) than most cereal crops. Quinoa is also rich in active ingredients, such as polyphenols, flavonoids, saponins, polysaccharides, peptides, and ecdysone, which provide balanced nutrition, enhance the body function, regulate blood sugar, decrease blood lipid, increase anti-oxidation and anti-inflammatory action, and prevent and treat cardiovascular diseases. Thus, quinoa is especially suitable for people suffering from chronic diseases, such as diabetes, hypertension, hyperlipidemia, and heart disease, and for the elderly people. Because of its comprehensive nutritional value and edible functional characteristics, quinoa is better than most grains and has become a highly nutritious food suitable for human consumption. This article reviews the active ingredients and physiological functions of quinoa, aiming to provide a reference for further research and its utilization in food, healthcare, and pharmaceutical research and development.

## 1 Introduction

*Chenopodium quinoa* Willd. (*Chenopodium quinoa* Willd.) is an annual herbaceous crop of the genus *Chenopodiaceae*. It is native to the middle and high-altitude mountains of Colombia, Ecuador, and Peru and to the Andes Mountains of South America. Similar to rice, it has a history of more than 6,000 years (1, 2). The nutritive potential of quinoa was rediscovered in the second half of the 20th century. The protein content of this crop is approximately 16–18%, which is high-quality protein and gluten-free. People with celiac disease or other digestive diseases can consume quinoa as a nutritional supplement (3). Quinoa is not only rich in dietary fiber, vitamins, and minerals but also contains all the essential fatty acids, amino acids, and bioactive compounds such as polyphenols, flavonoids, saponins, betaine, and phytosterols, which are beneficial to human health (4). A number of studies have shown that quinoa can prevent cancer, inflammation, hyperglycemia, hyperlipidemia, and other diseases and contribute to weight loss, anti-aging, etc. Quinoa is a typical example of a functional food that plays a role in promoting health (5). Because of its rich nutrition, the Food and Agriculture Organization of the United Nations recognizes that quinoa is the only monomer plant that can meet the basic nutritional needs of the human body. Quinoa is officially recommended as the most suitable perfect “whole nutritional food” for human beings and is listed as one of the ideal foods for astronauts in space missions by the United States Space Agency as well as one of the top 10 healthy and nutritious foods in the world (6).

In recent decades, due to lifestyle changes, chronic diseases have gradually become a major problem affecting modern populations. In order to prevent chronic diseases, people have begun consuming nutritious and functional foods to maintain body health. Quinoa is easy to cook and easy to digest and has a unique taste, supplementing nutrition, enhancing body function, regulating immunity and endocrine, preventing diseases, and so on. It meets people’s needs for nutrition, health, and safety and can be consumed by all groups. It is especially suitable for patients with chronic diseases such as hyperglycemia, hypertension and hyperlipidemia, and cardiovascular disease as well as for infants, pregnant women, the elderly, and other special physical groups (7). These functions are closely related to its basic nutrients such as protein, dietary fiber, vitamins, and various active substances. A number of studies have shown that quinoa contains polyphenols, flavonoids, saponins, and bioactive molecules, which possess anti-inflammatory and anti-cancer properties. These compounds can help lower cholesterol levels and reduce the risk of diabetes (8). Quinoa has attracted extensive attention from consumers and researchers due to its remarkable advantages in supplementing nutrition and maintaining health. Therefore, this paper aims to summarize the nutritional components and the efficacy of active substances in quinoa and to explore its physiological functions. The goal is to provide a reference for the full utilization of quinoa and to support further basic and clinical research.

## 2 Effect of quinoa on cardiovascular disease

Cardiovascular disease (CVD) is the primary cause of death and disability in the world, and diet is one of the most important risk factors (9). Some of the risk factors for CVD can be modified through lifestyle changes. Overweight, hypertension, and dyslipidemia are clinically recognized as the most important known risk factors for CVD. Many epidemiological studies have shown that a diet rich in whole grains is associated with reduced risk of cardiovascular disease (CVD) and mortality. A meta-analysis (10) on the effect of quinoa seed on cardiovascular disease (CVD) risk factors involving five eligible RCTs showed that quinoa seed supplementation significantly lowered body weight, waist circumference, fat mass, insulin serum levels, triglycerides (TG), total cholesterol (TC), and low-density lipoprotein (LDL) levels.

### 2.1 Decrease blood lipid

Hyperlipidemia is a common metabolic disease, which is an important inducing factor of atherosclerosis, coronary heart disease and other cardiovascular and cerebrovascular diseases, and diabetes mellitus. Quinoa oil is rich in fatty acids, 85.25% of which are unsaturated fatty acids (UFA). Pereira et al. (11) and Ayseli et al. (12) analyzed the fatty acid composition in quinoa oil and found that the contents of palmitic acid (20–21%), oleic acid (32–33%), and linoleic acid (27–31%) were relatively high. The total amount of monounsaturated fatty acids (MUFA) in quinoa was the highest. A diet high in MUFA can effectively control chylous disease, non-alcoholic fatty liver disease, and diabetes mellitus (11). Modern studies (13) have shown that the unsaturated fatty acids in quinoa can reduce low-density lipoprotein cholesterol (LDL-C) and increase high-density lipoprotein cholesterol (HDL-C), which can effectively prevent atherosclerosis.

#### 2.1.1 Population study on the effect of quinoa on blood lipid

Clinical trials (14) have shown that 35-year-old overweight women who consume 25 g quinoa powder daily for 4 weeks have a significant decrease in serum triglycerides (TG) and total cholesterol (TC) levels, while glutathione (GSH) has shown a significant increase. As an essential fatty acid, linoleic acid, which is rich in quinoa, can effectively reduce the lipid level of human muscle cells and can control inflammation and atherosclerosis by reducing the content of low-density lipoprotein cholesterol (LDL-C) in human blood.

A study by Navarro-Perez et al. (15) investigated the dose-dependent effect of quinoa seeds on reducing serum triglycerides in overweight and obese adults. A daily consumption of 50 g of quinoa was shown to reduce serum triglyceride levels in obese adults. The study team measured lipid distribution, body composition, and nutrient intake for 12 weeks in adults and controls who consumed quinoa seeds (25 g or 50 g/day). This study found that serum triglycerides were significantly lower in participants who consumed 50 g of quinoa seeds per day, although other biomarkers such as total cholesterol, HDL-C, and LDL-C did not change. High serum triglycerides are often a risk factor for cardiovascular disease (CVD), so the 36% reduction noted in this study is a positive sign that quinoa consumption has the potential to reduce CVD risk.

Another study (16) aimed to confirm the lipid-lowering effects of quinoa. Participants consumed quinoa cereal bars daily for 30 days, which led to significant reductions in triglycerides, total cholesterol, and low-density lipoprotein cholesterol levels. Additionally, there were modest reductions in body weight, blood pressure, and blood glucose levels. Another randomized crossover study (17) observed the effect of quinoa biscuits on cardiovascular disease biomarkers over a 4-week period. In this study, conducted with healthy older adults, researchers found a decrease in total and LDL cholesterol, TC:HDL ratio, body weight, and BMI in the quinoa group compared to the control group. All of these may contribute to lower CVD risk in older adults. However, there were no differences between the groups in changes in triglycerides, HDL cholesterol, PUFA or CRP concentrations, antioxidant status (FRAP), or BP.

A meta-analysis conducted by Atefi et al. (18) also showed that in adults, supplementation with quinoa at doses greater than 50 g reduced the risk of CVD by lowering serum triglycerides. Although the above studies showed modest changes in biomarkers associated with CVD, the mechanisms underlying this effect remain unclear. The authors also concluded that the lipid-lowering mechanism of quinoa mainly included the following: sterol inhibited the absorption of lipids, increased the clearance rate of lipids, and increased bile acid excretion; PUFA decreased the synthesis of lipids, increased the excretion of natural sterols, and changed the composition of fatty acids in lipid membranes. Phytosterols inhibited intestinal absorption of lipids and increased bile acid excretion.

#### 2.1.2 Animal studies on the effect of quinoa on lipids

A study (19) has found that *ω*-3 fatty acids in quinoa have vasodilatory and lipid-lowering effects, and its plant sterols (sitosterol, rapeseed sterol, stigmasterol, etc.) have physiological effects of antioxidant and lowering cholesterol. An *In vitro* study (20) showed that quinoa rutin can reduce vascular permeability and brittleness, dilate coronary arteries, and prevent hemagglutination, thus effectively reducing the risk of cardiovascular diseases. In addition, Yu et al. (21) also found that rutin in quinoa has multiple physiological effects, which can reduce the permeability and fragility of vascular smooth muscle cells in diabetic mice, prevent blood cell agglutination, dilate coronary arteries, and enhance coronary blood flow, thus preventing and treating cardiovascular diseases.

Through animal experiments, Paśko et al. (22) found that after feeding Wistar rats a high-sugar diet containing 310 g/Kg quinoa starch for 5 weeks, there were significant reductions in serum total cholesterol (26%), low-density lipoprotein (57%), triglycerides (11%), blood glucose levels (10%), and plasma total protein levels (16%). Additionally, quinoa starch reduced many adverse effects of a high-glucose diet on blood lipid and blood glucose levels. Through an in-depth study on the lipid-lowering effect of quinoa, Hu et al. (23) found that non-starch polysaccharides of quinoa were the main active components of lowering lipids. When the daily dose reached 5–10 g/Kg, the serum triglycerides, total cholesterol, and low-density lipoprotein contents of rats were significantly lower than those of the model group after 1 month of continuous administration. Cao et al. (24) studied the effect of quinoa soluble polysaccharides on blood lipid reduction in rats fed on a high-fat diet and found that after 8 weeks of oral administration of quinoa polysaccharides, the white fat of rats was significantly reduced. Additionally, the high dose [600 mg (/ Kg ·d)] was was more effective than the low dose [300 mg (/Kg ·d)]. After supplementation of quinoa polysaccharides, serum triglycerides and low-density lipoprotein cholesterol levels were significantly reduced in rats fed with a high-fat diet.

#### 2.1.3 Mechanism of quinoa in lowering blood lipids

A variety of functional substances in quinoa, such as flavonoids, polyphenols, and polysaccharides, have lipid-lowering effects. Noratto et al. (25) also chose fat diabetic mice as an experimental object and found that compared to the control group, quinoa intake can obviously reduce the mice’s plasma total cholesterol, low-density lipoprotein cholesterol, and oxidized low-density lipoprotein and can improve fatty liver disease. It is speculated that some proteins in quinoa can inhibit the synthesis of cholesterol, and the fiber in quinoa can inhibit the absorption of dietary cholesterol. Flavonoids in quinoa bran have a certain cholesterol-lowering effect on the liver of mice. This specific mechanism needs further study.

Quinoa has the effect of lowering blood lipids, partly because the extract can inhibit the activity of related enzymes or the expression of RNA. Takao et al. (26) showed that the protein component of quinoa could help prevent the increase in plasma and liver cholesterol levels because it inhibited the mRNA expression of key enzymes of cholesterol biosynthesis and promoted the mRNA expression of cholesterol catabolic enzymes in the liver. Xu (27) found that the peptides obtained from enzymatic digestion of quinoa protein had better cholate adsorption effect, indicating that it had better antilipidemic activity *in vitro*. Moreover, the peptides of quinoa polypeptide with a molecular weight of approximately 1,000 Da and a terminal residue of arginine may be an important factor for its good antilipidemic activity *in vitro*. The main sources of peptides with antilipidemic activity may be 11S seed storage globulin and N-glycosidase protein of quinoa protein. These studies provided evidence that quinoa has the effect of lowering blood lipids.

### 2.2 Antihypertension

Hypertension is an important risk factor that increases the risk of cardiovascular disease. Bioactive peptides are mixtures of free amino acids and low molecular weight peptides of different chain lengths (220 amino acid residues) released by proteins under physiological conditions as a result of gastrointestinal enzymes. Small peptides are released from proteins due to the action of digestive enzymes such as pepsin, trypsin, chymotrypsin, and peptidase.

In addition to their functions in regulating important physiological processes such as lowering blood lipids, reducing blood glucose, and providing anti-oxidant effects, bioactive peptides also play an important role in preventing hypertension. Isolated dietary protein-derived peptides have been shown to have antihypertensive activity by affecting various molecular mechanisms, including inhibition of angiotensin-converting enzyme (ACE), reduction in systolic blood pressure, reduction in angiotensin II levels and AT1R expression, enhancement of vasodilation, improvement of central blood pressure and arterial stiffness, and inhibition of vasoconstriction through PPAR-*γ* expression (28–31).

Guo et al. (32) showed that quinoa peptide obtained through *in vitro* digestion showed a hypotensive effect in the form of ACE inhibitory activity in rats. One peptide, QHPHGLGALCAAPPST, identified from the tryptic hydrolysate of quinoa chyme, inhibits ACE by binding to the number of active hot spots of the ACE enzyme. Three potential bioactive peptides, FHPFPR, NWFPLPR, and NIFRPF, were further investigated, and their inhibitory effects on ACE were confirmed. Molecular docking studies provided a new perspective on the binding of ACE to peptides and revealed that the presence of specific amino acids in the peptide sequence (Pro, Phe, and Arg at the C-terminus and Asn at the N-terminus) may contribute to the interaction between ACE and peptides.

Another *in vivo* study by Guo et al. (33) used quinoa protein to study spontaneously hypertensive rats (SHRs) for 5 weeks *in vivo*. After administration of quinoa protein, the blood pressure of rats significantly decreased, *α* diversity significantly increased, and the microbial structure changed to that of non-hypertensive rats. In addition, in quinoa protein-treated SHRs, blood pressure was highly negatively correlated with the increased abundance of Turicibacter and Allobaculum. Interestingly, the fecal microbiota of SHRs treated with quinoa protein shared more features in genus composition with non-hypertensive rats than did the captopril group. These results suggest that quinoa protein may serve as a potential candidate to lower blood pressure and ameliorate hypertension-related gut microbiota dysregulation.

Zheng et al. (34) investigated the *in vivo* antihypertensive effect of ACE inhibitory peptide and antioxidant peptide in quinoa bran albumin. Based on computer analysis, an ACE inhibitor and antioxidant peptide, RGQVIYVL, along with two other antioxidant peptides, ASPKPSSA and qflagr, were identified from QBAH. RGQVIYVL demonstrated a high ACE inhibitory activity with a competitive inhibitory mode, resulting in a significant antihypertensive effect in spontaneously hypertensive rats. The results of the molecular docking simulation showed that RGQVIYVL could interact with the active ACE site through hydrogen bonds with high binding force.

Another study (35) of the effect of quinoa yogurt on blood pressure showed that dietary protein peptides of quinoa yogurt drink fermented by 21 strains of probiotic lactic acid bacteria had protective effects on diabetes and hypertension. In this study, QLCZ had the strongest inhibitory effect on ACE. Among the strong inhibitory peptide sequences found, LAHMIVAGA and VAHPVF showed significant *α*-glucosidase and ACE inhibitory activities. Therefore, the protein hydrolysates and peptides of quinoa yogurt have the potential ability to regulate blood pressure.

### 2.3 Antioxidant

Oxidative stress is associated with various diseases with the pathogenesis of inflammation. Reducing oxidative stress is helpful for the treatment of atherosclerotic heart disease. The antioxidant activity of quinoa is caused by several factors. First, the saponins, polysaccharides, flavonoids, and phenolic acids contained in quinoa (36–38) have good antioxidant activity. Second, the antioxidant peptides released by quinoa protein during digestion (39) also have good biological activities. Finally, ascorbic acid, sterol, tocopherol, and other substances contained in quinoa (11) have antioxidant properties. An *in vivo* experiment (40) of spontaneously hypertensive rats revealed the antioxidant effect of red quinoa hydrolysate and its ability to ameliorate hypertension and its associated complications.

The extracts of quinoa seeds, buds, and leaves are high in polyphenols, which are good antioxidants *in vitro* and can be used as excellent antioxidant food (41). The phenolic compounds metabolized by quinoa (flavonoids, phenolic acids, and lignins) can be used as free radical scavengers and reducing agents, which can inhibit the oxidation reaction caused by free radicals. Studies have found that 80% ethanol extract of quinoa can significantly scavenge ABTS and DPPH free radicals *in vitro* (42). Gawlik-Dziki et al. (43) found in experiments that the high content of phenolic substances in quinoa makes it possess good antioxidant properties, including reduction, free radical scavenging, metal chelation, lipid antioxidant, and other abilities. After digestion and absorption of quinoa *in vivo*, it can reduce the concentration of malondialdehyde in plasma and enhance the activity of antioxidant enzymes. It has been proved that quinoa may have certain efficacy in the prevention of inflammatory diseases and other diseases related to oxidative stress.

Relevant studies (44) have shown that quinoa polysaccharide is an effective free radical scavenger. Hu et al. (45) successfully isolated from quinoa seed a new polysaccharide composed of glucose and galactose aldehyde with a molecular weight of 8,852 Da. This kind of low molecular weight polysaccharides *in vitro* showed significant oxidation resistance and immune regulation effect, which provided a theoretical basis for quinoa polysaccharides for the prevention of oxidative stress-related diseases.

Quinoa saponins are mainly distributed in seed coat and bran and have strong antioxidant activity (46). Studies (47) have found that the saponins in quinoa can also reduce cholesterol, regulate substance metabolism, induce changes in intestinal permeability, and promote absorption of specific drugs. Zhang et al. (48), using a combination of metabolomics and intestinal flora analysis, confirmed that high doses of quinoa saponins change the levels of glycosides, L proline, and other energy metabolism substances in rat urine. These saponins also affect the level of amino acids in the urine of rats and the metabolism of vitamin B6, ammonia circulation, and the tryptophan metabolism pathway, thereby regulating the metabolism of the body. This, in turn, influences its intestinal flora distribution and changes the intestinal microenvironment. With the increase in vitamin B6, the degradation of homocysteine slows down, which can harm the cardiovascular health of rats (49), and the upregulation of L-serine also slows down the degradation of homocysteine.

Flavonoids are strong biological antioxidants that help scavenge oxygen free radical in the body. In addition to their antioxidant properties, they also contribute to lowering cholesterol and improving blood circulation from a medical perspective. The seeds and leaves of quinoa are rich in flavonoids (50, 51). The main flavonoids in leaves are quercetin and kaempferol (52, 53). Studies have shown that the types of flavonoids in quinoa seeds are directly related to seed color, and the darker the seed coat color, the higher the content of flavonoids and the stronger the antioxidant activity of quinoa seeds (54).

**Figure 1:**
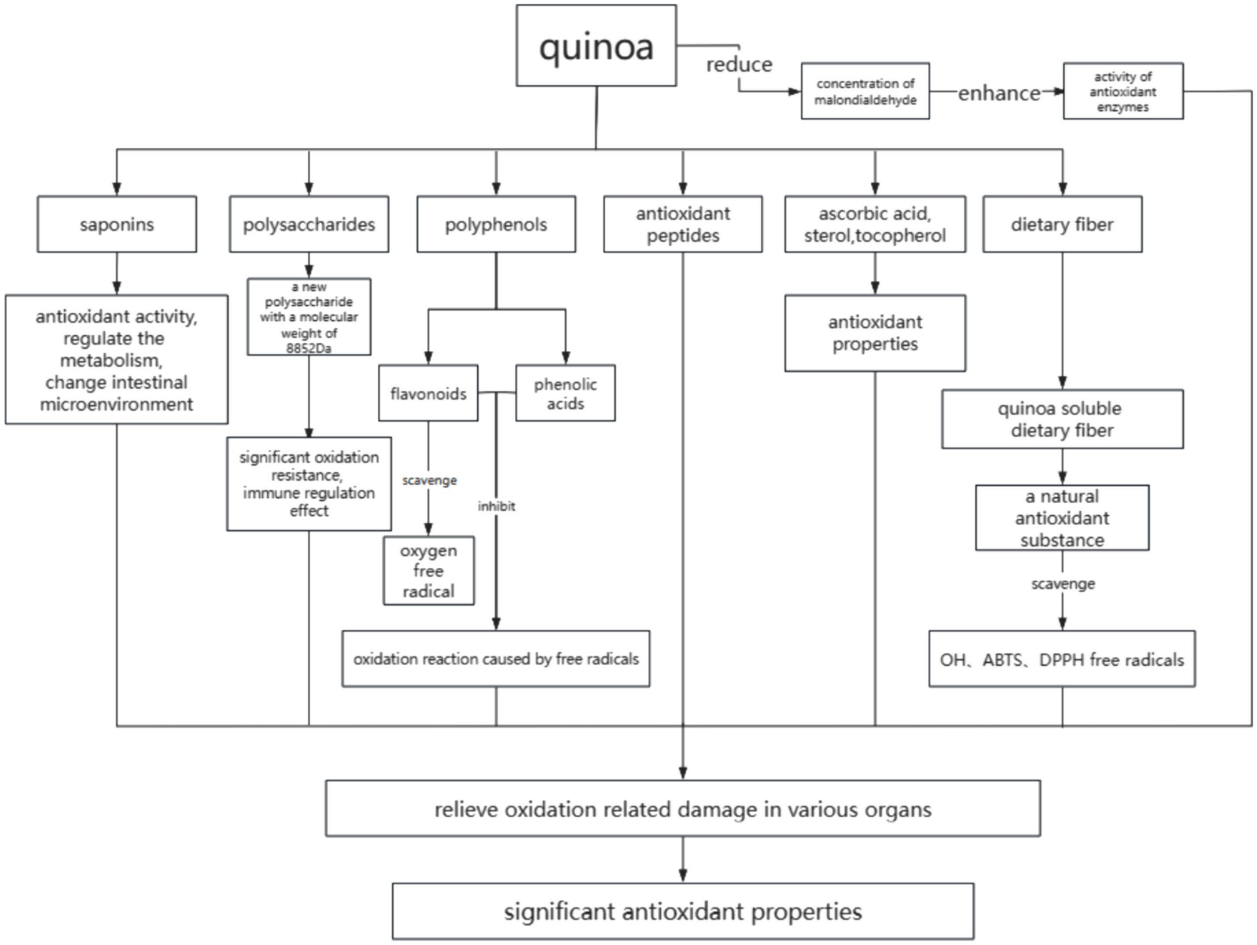
Mode of action of antioxidant activity of quinoa.

Dietary fiber refers to a class of carbohydrates that cannot be hydrolyzed by endogenous enzymes in the human small intestine but can be partially fermented and utilized by some microorganisms in the large intestine (55). It can be divided into soluble dietary fiber (SDF) and insoluble dietary fiber (IDF). The soluble dietary fiber plays an important physiological function in improving intestinal flora, preventing gastrointestinal diseases, regulating postprandial blood glucose, and preventing chronic diseases such as cardiovascular and cerebrovascular diseases (56–59). The quinoa soluble dietary fiber is a natural antioxidant substance. Wang et al. (60) studied the antioxidant activity of soluble dietary fiber extracted from quinoa. The results showed that the soluble dietary fiber had good scavenging ability on OH, ABTS, and DPPH free radicals (Figure 1; Table 1).

**Table 1:** Benefits of functional composition of quinoa on cardiovascular disease.

Cardiovascular disease		
Decrease blood lipids	Antihypertension	Antioxidants
Flavonoids, polyphenols, polysaccharides, monounsaturated fatty acids (MUFA)	Bioactive peptides	Saponins, polysaccharides, flavonoids, phenolic acids, dietary fiber

## 3 Effect of quinoa on diabetes mellitus

Diabetes has always been a common chronic disease, threatening human health. In addition to conventional drug treatment of diabetes, nutritional treatment, especially choosing foods with a low glycemic index (GI), is a key measure for controlling blood glucose in patients with diabetes. Antioxidants found in quinoa, including vitamin C, vitamin E, saponins, polyphenols, and flavonoids play an important role in reducing postprandial blood glucose levels, blood lipids, and cardiovascular disease risk, as well as providing anti-inflammatory benefits for patients with diabetes. A recent meta-analysis (61) showed significant evidence of a non-linear association between intervention and fasting blood glucose (FBG) based on the quadratic model. The effect of quinoa on blood glucose regulation occurs mainly through the slow release of glucose, reducing the burden of insulin secretion by the pancreatic islet cells, promoting the activity of islet cells, and improving insulin resistance, glucose metabolism, intestinal microbiota, and so on.

### 3.1 Research status of the effect of quinoa on blood glucose in patients with diabetes mellitus

Quinoa is a food with a low GI index (62), and dietary intervention of quinoa is an effective means to regulate blood glucose in patients with diabetes. The glycemic index (GI) of quinoa is 53 ± 5, much lower than that of rice (69 ± 7) and wheat (70 ± 5) (63), which can delay the rise of blood glucose and achieve the purpose of lowering blood glucose. Quinoa has higher levels of xylose and maltose and lower levels of glucose and fructose. This composition contributes to the slow release of glucose into the body, helping to control the blood sugar balance and reduce the feeling of hunger. By avoiding a peak in blood glucose levels 2 h postprandially and promoting a gradual rise in blood sugar, quinoa helps compensate for insufficient insulin secretion from islet beta cells or prevents postprandial hyperglycemia. Consequently, it reduces insulin secretion, thus reducing the burden on the pancreas. The remaining islet *β* cells are placed in a state of rest, and the β cells create a resting environment to promote the recovery of insulin production and reserve function of the remaining β cells. At the same time, the slow release of glucose provides the body with a stable, continuous source of energy for a long period, reducing the risk of hypoglycemia (64). Wang et al. (65) found that after 12 days of quinoa and rice mixed diet intervention, the 2-h postprandial blood glucose levels of diabetic patients in the observation group were significantly lower than that in the control group, indicating that the quinoa diet was helpful to alleviate the rise of postprandial blood glucose levels. Gabrial et al. (66) found that the blood glucose levels of patients began to decrease steadily after eating quinoa for breakfast and finally returned to below their fasting levels. Compared to white wheat bread, the blood glucose concentration before the second meal was significantly lower after the quinoa breakfast intake, indicating the potential health benefits of quinoa in improving glucose tolerance at the first and second meals. Fang (67) also found that compared to the control group, the observation group of diabetic patients who consumed quinoa steamed bread had a smaller increase in blood glucose levels 2 h after the meal. These results indicated that low glycemic index food (mainly quinoa) supplemented with insulin therapy can more effectively control hyperglycemia in type 2 diabetes mellitus, especially postprandial blood glucose levels. This approach can significantly reduce the amount of insulin required and shorten the treatment time of patients and does not increase the incidence of hypoglycemia.

### 3.2 Current research status of the effect of quinoa on blood glucose in diabetic mice

Studies on regulating the blood glucose of quinoa in diabetic mice have shown that quinoa plays a significant role in reducing blood glucose. Liu et al. (68) established a diabetic mouse model and found that the fasting blood glucose levels of mice fed with quinoa flour decreased after 6 weeks of feeding. Compared to the mice without a quinoa meal, the area under the blood glucose curve of the mice fed with quinoa meal decreased in the oral glucose tolerance test, and the decrease was more obvious in the high dose group, and the effect of lowering blood glucose was better. Liu et al. (69) found that quinoa fermentation broth could reduce fasting blood glucose levels, triglycerides, and cholesterol levels in type 2 diabetic mice. Jiao (70) and Cai (71) both found that quinoa could increase the levels of liver glycogen and insulin and reduce the levels of fasting blood glucose and fructosamine in mice, and the hypoglycemic effect was more obvious, especially in the high-dose quinoa group. The function of islet cells had improved, and insulin secretion had increased. Li (72) found that quinoa intervention significantly reduced fasting blood glucose in mice, enhanced glucose tolerance, improved insulin resistance, improved the structure of intestinal flora in mice, and increased intestinal probiotics.

### 3.3 Mechanism of quinoa on blood glucose regulation

#### 3.3.1 Slow release of glucose to maintain blood glucose stability in the body

Quinoa is a low GI food, which has a slow absorption rate of carbohydrates, a long residence time in the gastrointestinal tract, a slow release of glucose, and a low peak value of glucose after entering the blood. Liu et al. (68) showed that administration of quinoa powder (54 g/100 g) in diabetic mice could significantly reduce the level of fructosamine in mice, suggesting that quinoa could reduce blood glucose levels and promote blood glucose homeostasis.

Dietary fiber is negatively correlated with the risk of type 2 diabetes, and increasing dietary fiber intake can significantly improve the blood glucose level of patients with type 2 diabetes (73). A recent RCT (74) showed that adding quinoa to staple food intake can reduce postprandial blood glucose and improve lipid metabolism and insulin resistance, delaying the progression of diabetes in people with impaired glucose tolerance. Another study (75) has that the dietary fiber of quinoa is mostly insoluble fiber, composed of galactose, galacturonic acid, xylose, and glucose. Carbohydrate dietary fiber can not only slow food digestion and absorption of carbohydrates and increase satiety by inhibiting the activity of hydrolytic enzymes but also can increase the viscosity of the liquid in gastrointestinal liquids. This increased viscosity inhibits glucose absorption in the small intestine, prolongs food residence time in the stomach, and slows the gastric emptying rate, As a result, the absorption rate of glucose through the intestinal wall is reduced, thus achieving the purpose of improving postprandial blood glucose levels (76, 77).

Some functionally active molecules in quinoa can also play a role in lowering blood glucose. Zhou et al. (78) found that peptides generated by enzymatic hydrolysis of quinoa and glutamic acid in the amylase active site were bound by intermolecular force, which resulted in the decrease of amylase activity, which explained the reason for the low glycemic index of quinoa. Hemalatha et al. (79) reported that phenolic substances in quinoa have obvious inhibitory effects on *α*-amylase and α-glucosidase activities, which can help delay the absorption of dietary carbohydrates in the human body. Polyphenols in quinoa exist in the form of free phenols and bound phenols, and both forms of polyphenols have certain hypoglycemic activities. Han et al. (80) studied the functional activities of the collected quinoa samples and found that both the free and bound phenolic extracts of quinoa had high inhibitory activities on *α*-glucosidase, among which three free phenolic extracts of quinoa showed higher inhibitory effects than acarbose. These results indicated that these three quinoa varieties had a potential role in controlling hyperglycemia and could help the human body inhibit the absorption of glucose in the small intestine. Tang et al. (81) demonstrated through enzyme inhibition experiments *in vitro* that phenols and flavonoids in quinoa could inhibit the activities of *α*-amylase, *α*-glucosidase, and pancreatic lipase, reduce the digestion and absorption of starch and triglyceride, and prevent the glycolipid conversion process. Herrera et al. (82) evaluated the inhibitory activities of quinoa extract on pancreatic lipase and *α*-amylase by traditional *in vitro* methods and simulated intestinal digestion, and the results showed that quinoa extract could inhibit the activity of pancreatic lipase. Moreover, it also had a slight inhibitory effect on α-amylase. Therefore, quinoa was considered to be the preferred cereal raw material substitute for inhibiting carbohydrate digestion and regulating the glycemic index.

Quinoa seed extract contains 20-hydroxyl peeling hormone, phytosteroids, flavonoid glycosides, oils, and proteins, which can significantly reduce fasting blood glucose (FBG) in obese diabetic mice. It may be that 20E itself has a strong effect on lowering blood glucose levels and preventing obesity, and other active ingredients in the leaching solution, such as flavonoids, fatty acids, and amino acids, can cooperate with 20E to enhance its effect on lowering blood glucose levels (19). 20-hydroxyecdysone (20E) is one of the most important phytodesquamate sterols in plants, which possesses good antioxidants, blood glucose-lowering, and obesity-inhibiting effects (83). Kizelsztein et al. (84) also demonstrated that quinoa ecdysone has the effect of lowering blood glucose and anti-obesity. In the culture of murine liver cancer cells (H4IIE), it was found that 20-hydroxy ecdysone (20E) reduced the expression of phosphoenolpyruvate kinase (PEPCK) and glucose 6 phosphatase (G6Pase), decreased the content of glucose, and induced the sensitivity of Akt2 phosphorylation to phosphoserine 3 kinase-specific inhibitor LY-294002, which could reduce blood glucose and blood lipids. In addition, daily supplementation of 20-hydroxyecdysone (at a dose of 10 mg/Kg) to diet-induced obesity and insulin resistance C57BL/6 J rats for 13 weeks significantly reduced plasma insulin levels and glucose tolerance, as well as body weight and fat mass in obese rats.

#### 3.3.2 Reducing the secretion burden of islets and promoting islet cell activity

Blood glucose regulation plays a role by protecting and improving islet B-cell function and reducing islet secretion sharing. Studies (85) have shown that in diabetic mice fed with quinoa meal, the level of fructosamine in the body is significantly reduced, the plasma insulin level is increased, and the highest insulin level is induced by high-dose quinoa meal, suggesting that quinoa may improve the function of the islet B cells and increase insulin secretion. Quinoa is also rich in *ω*-3 polyunsaturated fatty acids (ω-3 PUFA), which is also important for increasing the activity of islet B cells (86). Quinoa contains less glucose and fructose, but more xylose and maltose, which can release glucose slowly in the body to avoid the rapid rise of postprandial blood glucose. The islet cells are overloaded with hyperglycemic signals to secrete insulin, thereby reducing insulin secretion and reducing the burden of insulin secretion by the pancreas (85).

#### 3.3.3 Improvement of insulin resistance

Insulin resistance is a major pathophysiological feature of type 2 diabetes mellitus, characterized by a decreasing ability of insulin to regulate glucose metabolism. This leads to an accumulation of glucose in the bloodstream because cells are unable to fully absorb it, leading to an increase in blood glucose levels and a compensatory increase in insulin secretion by the body (87). Selma-Gracia et al. (88) showed that quinoa bread reduced serum insulin levels and improved insulin resistance in hyperglycemic mice fed with a high-fat diet. Hu et al. (89), Purushotham et al. (90), and Wang et al. (91) suggested that the quinoa complex may regulate hepatic glycolipid metabolism and improve insulin resistance, hepatic gluconeogenesis, and fatty acid metabolism by upregulating the expression of SIRT1 and PGC 1 *α* proteins. Li (72) found that a quinoa diet could enhance blood glucose regulation and insulin sensitivity in mice. After quinoa intervention, the phosphorylation of IRS-1 (Ser307) decreased, and the phosphorylation of PI3K (Tyr458), AKT (Ser473), and GSK 3-*β* (Ser9) significantly increased. The protein expression of GLUT-4 was significantly increased. Quinoa dietary intervention may improve insulin metabolism signaling pathway and GLUT-4 protein expression by activating the PI3K/AKT signaling pathway and promoting GLUT-4 membrane translocation in the skeletal muscle of mice and then improve anti-diabetic treatment at the protein level.

#### 3.3.4 Improving glucose metabolism

Glucose metabolism disorder refers to the abnormal structure, concentration, and function of hormones or enzymes that regulate the metabolism of glucose, fructose, and galactose in the body, or the lesions of related tissues and organs, resulting in excessive or insufficient blood glucose in the body. The starch content in quinoa is low, which helps regulate glucose metabolism, inhibit, or activate glycosidase reactions, and regulate abnormal blood glucose levels. The high content of dietary fiber in quinoa can significantly reduce the sensitivity of digestive enzymes and slow down the growth of starch digestibility after a certain period of time. In a recent experimental study (92), red quinoa polysaccharides (RQP) showed good antioxidant activity and *α*-amylase and α-glucosidase inhibitory activity *in vitro* and could inhibit the development of diabetes by correcting the imbalance of intestinal flora. Specifically, the supplementation of RQP improved the antioxidant function of diabetic mice, reducing inflammation and promoting the production of SCFAs.

#### 3.3.5 Improvement of gut microbiota

Quinoa has the potential to regulate gut microbiota and promote gut health. A study (93) has shown that the consumption of quinoa can alleviate the intestinal microbial dysregulation induced by sodium dextran sulfate in mice, reducing the clinical symptoms caused by it and reducing the disease activity index and the degree of tissue damage. Lamothe et al. (94) found that arabglycan and pectin polysaccharides in quinoa have the effects of regulating intestinal microbiota, assisting to protect gastric mucosa and anti-ulcer. Another study (95) showed that the extract of quinoa has a bacteriostatic effect, which can increase the growth of general bacteria, such as Bifidobacterium and Lactobacillus, and inhibit the growth of Proteobacteria. Another experimental study (96) suggested that quinoa could regulate the microflora disorder in diabetic mice, which was induced by a high-fat diet combined with streptozotocin, and has the effect of alleviating hyperglycemia. The prediction of network pharmacological results showed that quinoa may exert hypoglycemic effects through gut microbiota and the TAS1R3/TRPM5 taste signaling pathway. Li (72) also found that the diversity and evenness of intestinal microbiota in mice after quinoa intervention were improved. In the intestinal microbiota of mice in the quinoa group, the increase in Parasutterella and Muribaculum in the quinoa group may reflect the improvement of glucose tolerance and metabolic disease symptoms in mice. The increased levels of Faecalibaculum and *Lactobacillus reuteri* may reflect a healthy intestinal microbiota structure (Table 2).

**Table 2:** Mechanisms of quinoa on regulating blood glucose in diabetes mellitus.

Diabetes mellitus	
Slow release of glucose to maintain blood glucose stability in the body	Xylose and maltose, dietary fiber, phenols and flavonoids, 20-hydroxyl peeling hormone, phytosteroids, flavonoid glycosides
Reducing the secretion burden of islets and promoting islet cell activity	
Improvement of insulin resistance	
Improving glucose metabolism	
Improvement of gut microbiota	

## 4 Conclusion

Quinoa has high nutritional value. As a characteristic multigrain nutritional food and functional health food, quinoa has significant benefits. Its biological functions are mainly reflected in antioxidant, antibacterial, and anti-cancer activities, as well as lowering blood glucose and cholesterol levels. In recent years, various studies have found that its bioactive components (polyphenols, flavonoids, saponins, and polysaccharides) have a variety of pharmacological effects. This has improved its application value in areas such as food, medical care, and drug development. With the increasing awareness and demand for healthcare in the modern population, the application of quinoa nutrition and health products has a good prospect. Future research should focus on elucidating the mechanisms underlying the biological activity of quinoa’s functional ingredients, such as polyphenols, flavonoids, saponins, peptides, polysaccharides, and the molting hormone. This includes investigating their efficacy and regulatory functions within the human body as well as in genomics, proteomics, metabolomics, and other fields of study, thereby boosting the nutrition and healthcare value of quinoa in human chronic diseases and treatment to the maximum.
