# Corrigendum: Effects of quinoa on cardiovascular disease and diabetes: a review

**DOI:** 10.3389/fnut.2024.1516854

**Published:** 2024-11-18

**Authors:** He Zhang, Ruiqi Li

**Affiliations:** ^1^Xiyuan Hospital, China Academy of Chinese Medical Sciences, Beijing, China; ^2^National Clinical Research Center for Chinese Medicine Cardiology, Beijing, China; ^3^The Third Affiliated Hospital of Beijing University of Chinese Medicine, Beijing, China

**Keywords:** quinoa, cardiovascular disease, diabetes, efficacy, review

In the published article, there was an error in the author list. The author order list was reversed, the wrong author was listed as the corresponding author and one of the authors' name was reversed. The error read as:

Li Ruiqi 1^†^ and He Zhang2,3^*†^

A correction has been made to the author order list. It should read as:

“He Zhang1,2^†^ and Ruiqi Li3^*†^” with Ruiqi Li listed as the corresponding author, ruiruiqiqi1993@hotmail.com.

The authors apologize for this error and state that this does not change the scientific conclusions of the article in any way. The original article has been updated.

